# Synthetic Biology Based on Genetic Logic Circuit, Using the Expression of Drug Resistance, BCRP Pump in MCF-7 Cancer Cell Line

**DOI:** 10.22037/ijpr.2020.111970.13459

**Published:** 2020

**Authors:** Marzieh Gerami, Modjtaba Emadi-Baygi, Mohammad Eshghi, Fatemeh Elahian, Razieh Heidari, Mehdi Hosseinzadeh

**Affiliations:** a *Department of Computer Engineering, Science and Research Branch, Islamic Azad University, Tehran, Iran. *; b *Department of Genetics, Faculty of Basic Sciences, Shahrekord University, Shahrekord, Iran. *; c *Research Institute of Biotechnology, Shahrekord University, Shahrekord, Iran. *; d *Department of Computer Engineering, Faculty of Computer and Electrical Engineering, Shahid Beheshti University, Tehran, Iran. *; e *Department of Medical Biotechnology, School of Advanced Technologies, Shahrekord University of Medical Sciences, Shahrekord, Iran. *; f *Institute of Research and Development, Duy Tan University, Da Nang 550000, Vietnam. *; g *Mental Health Research Center, Psychosocial Health Research Institue, Iran University of Medical Sciences, Tehran, Iran.*

**Keywords:** Synthetic biology, Logical circuits, MCF-7 Cancer cell line, BCRP, Lentiviral vectors

## Abstract

Biological circuits are developed as biological parts within a cell to carry out logical functions resembling those studied in electronics circuits. These circuits can be performed as a method to vary cellular functions, to develop cellular responses to environmental conditions, or to regulate cellular developments. This research explored the possibility of synthetic biology based on the genetic logic circuit *A* and (not *B*) using the inducible expression of the both BCRP drug resistance pump and its specific shRNA in MCF-7 cancer cell line utilizing the third generation of lentiviral vectors. The accuracy of the output of the proposed circuit for living cells, was confirmed by the results of the Real-Time PCR and flow cytometry at the RNA and protein levels. At the RNA level, the effect of the inducers on the BCRP gene expression and silencing were investigated by real-time PCR. Furthermore, at the protein level, induction of the expression of the BCRP pump resulted in driving out of the substrate from inside the cells leading to the decrease of the fluorescent emission from the transfected cells. We successfully designed and implemented the genetic logic circuit *A *and (not *B*) using the inducible expression of the both BCRP drug resistance pump and its specific shRNA in MCF-7 cancer cells.

## Introduction

Synthetic biology is a combination of two areas of biotechnology and engineering science that is rapidly evolving. Modeling and analyzing biological systems based on engineering concepts for researchers is very attractive (1). The main applications of synthetic biology, are health and medicine (2-5), environment (6), biofuels (7, 8) and agriculture (9-15). 

The achievements that have been applied in synthetic biology are similar to those used in microelectronics. Today, a library of elements has been created in microelectronics, known as basic components. These components are connected to each other and create a complex system such as processors. Synthetic biology also shows the system as a biological component (bioparts). These components can ultimately become a system (16).

Each electrical circuit has a clear function and certain behavior. When the input voltage is applied to the circuit, the circuit will display the output response. Similarly, the gene regulating circuit will generate the output protein in the presence or absence of the input or concentration of the input proteins.

Electronic devices are controlled by binary calculations and using electrons that pass through metal wires and connect logic gates together. Similarly, genetic circuits are controlled by cellular and environmental signals, physiological activities, and metabolic calculations. Therefore, the goal is to focus on the development of biological circuits and their applications, so as to integrate VLSI technology and biological circuits. This achievement is similar to the design in the traditional VLSI but requires biology information obtained from the results of the experiments (17-20).

Biological circuits are developed as biological parts within a cell to carry out logical functions resembling those studied in electronics circuits. To alter cellular functions, establish cellular reactions to environmental conditions, or regulate cellular development, these circuits can be used as a method.

Scientists can employ living systems as engineered biological parts to achieve a broad range of valuable functions by implementing convenient logic elements in cellular systems. If the design of synthetic genetic circuits that behave like particular electrical circuits is possible, it can be said that genetic circuits have the potential to help us to better understand how microorganism function, produce drugs more economically, metabolize toxic chemical and even modify bacteria to hunt and kill tumors (21).

In this regard, the present study deals with one of the aspects of synthetic biology. The accuracy of the proposed idea for living cells has also been tested *in-vitro*. 

*Design of Biological Gates*

*Design of NOT, AND and NAND genetic gates*

**Figure 1:**
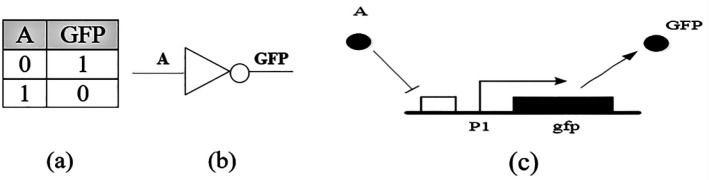
(a) Truth table of an inverter, (b) Schematic symbol, (c) Genetic circuit of a NOT gate

The simplest gate is the inverter gate or the NOT gate, Figure 1. This genetic circuit is made up of a fluorescent gene and a promoter suppressed by “*A*”. If “*A*” protein does not exist in the cell, then transcription of the gene is performed and the output turns ON. The “0” output implies the protein is not in an appropriate concentration, and “1” indicates that the protein is in an acceptable concentration (16).

**Figure 2:**
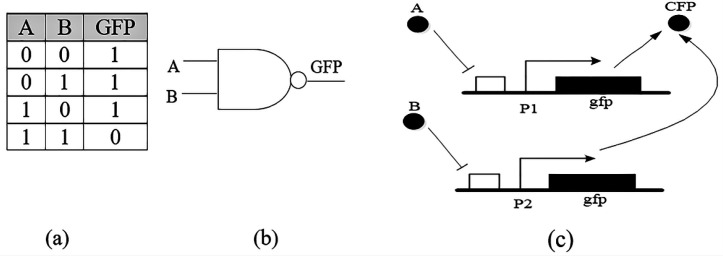
(a) Truth table of NAND gate, (b) schematic symbol, (c) Genetic implementation of NAND gate

The NAND gate with two inputs is shown in Figure 2. The inputs are two “*A*” and “*B*” proteins and a fluorescent compound as outflow and its behavior is based on the truth table, the fluorescent gene is expressed when there is no “*A*” or “*B*” in the cell (16).

*Logical Mask Gate*

Combinational logic circuit is a type of digital circuit which is implemented by Boolean functions, where the outputs are determined only by the status of present inputs. In order to make larger circuits in computer systems for various purposes, such as processing, logic gates are connected together to make new circuits. By joining the two gates which are the basis of logical algebra, described in section 1.1, the circuit according to the following figure, is obtained.

**Figure 3:**
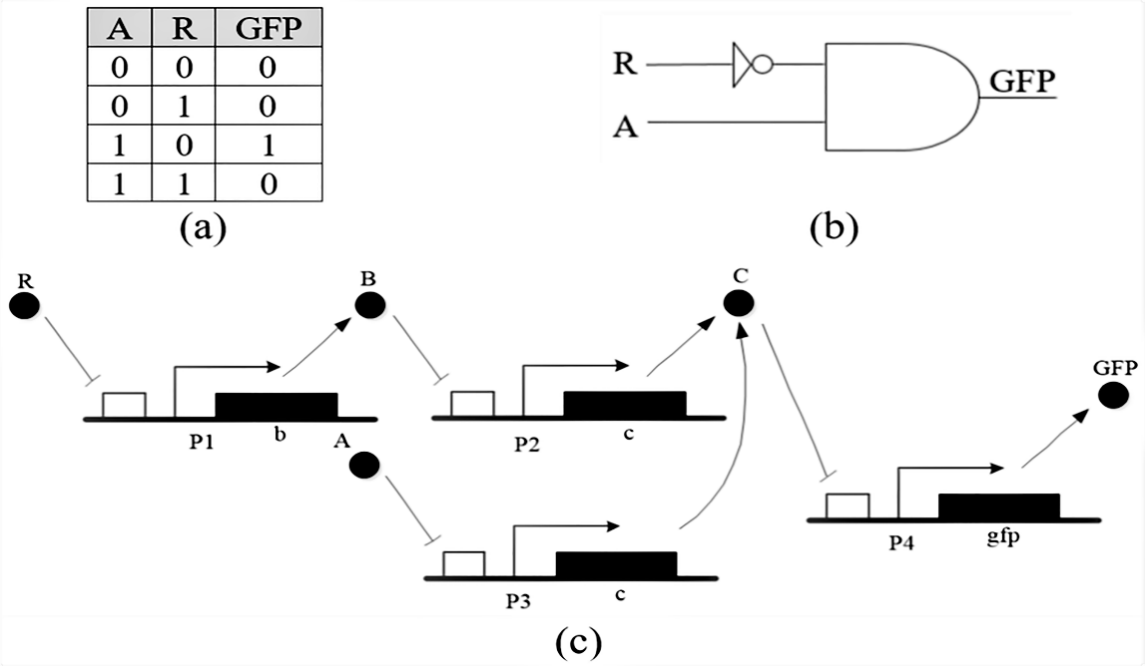
(a) Truth table of A AND NOT(R) circuit, (b) Schematic symbol, (c) Genetic circuit of A AND NOT(R)

Logical function of the circuit of Figure 3, gives the biological representation consisting of a fluorescent gene which can be activated by protein *A* and repressed by protein *R*.

*Resistance to treatment*

Overcoming the drug resistance over time is a major obstacle in cancer therapy because chemoresistance causes recurrence, metastasis and leading to death. Therefore, understanding the molecular mechanisms of resistance are very important and helpful for finding novel therapeutic approaches in cancer therapy (22). Transporter pumps are the molecular mechanisms of chemoresistance. ABC proteins are members of transport system for the translocation across cellular membranes using ATP-binding cassette transporter (23).

As a member of the ABC family, overexpression of breast cancer resistance protein (BCRP/ABCG2) may be observed in normal tissues (like the placenta, intestine, liver, blood-testis or brain barrier, hematopoietic progenitor and other stem cells) and chemoresistant cancer cells for the efflux of cytotoxic drugs such as mitoxantrone. ABCG2 was overexpressed in the presence of doxycycline in comparison to the parental cells (23).

In this paper, we aim to check the feasibility of biological mask gate, (*A* AND NOT(*R*)) to reduce drug resistance in MCF-7 cancer cells.

## Experimental

*Cell line and cell culture condition*

**Figure 4:**
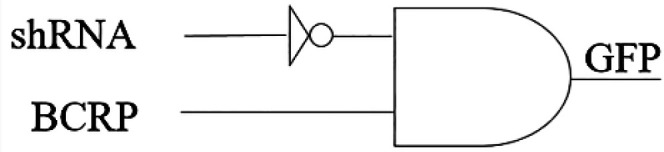
**Schematic of the proposed logic circuit with the aim of reducing drug resistance in MCF-7 cancer cells**

The proposed circuit inputs are considered in accordance with Figure 4. The fluorescent drug is pumped when BCRP input exists and another input, shRNA, does not exist. The accuracy of the proposed circuit for evaluation of BCRP gene expression (mRNA) was investigated by real-time RT-PCR. Doxycycline induced the expression level of BCRP gene in MCF-7 breast cancer cell line transfected with the corresponding vector. 

BCRP protein expression was determined flow cytometrically by measuring mitoxantrone accumulation in the absence and presence of the inhibitor, novobiocin.

As the result show, the proposed circuit resulted in the accumulation of the drug in the cancer cells and reduces drug resistance in the MCF-7 cells. Therefore, it could be of great importance to consider the combination of biology with microelectronic in finding or optimizing new treatments for breast cancer patients. We treated cells with sodium butyrate, a well-known inhibitor of histone deacetylases (HDAC), that causes histone hyperacetylation, chromatin decondensation, and activation of silenced promoters (24).

The doxycycline induction factor causes the transcription of the BCRP gene and its expression. The vectors used in this study were designed by the authors and ordered to be manufactured by GeneCust (Luxembourg).

Many viruses turn the host organism into a factory for the propagation of the virus by integrating their own genome into that of a host organism. This characteristic can be applied for genetic modification for basic research. Biomedical researches have exploited lentiviral vectors since the third generation vectors have a convincing safety to be used by biologists with minimal specialist training (25).

RNA interference (RNAi) is a natural process through which the expression of a targeted gene can be knocked down. A short hairpin RNA (shRNA) is an RNA molecule that can be applied to silence target gene expression via RNA interference. As an effective mediator of RNAi, shRNA has an approximately low rate of degradation and turnover. Furthermore, shRNA constructs have been used in low copy numbers allowing high potency and sustainable effects that result in less off-target effects. However, it requires use of an expression vector, which has the potential to cause side effects in medicinal application (26).

**Figure 5:**
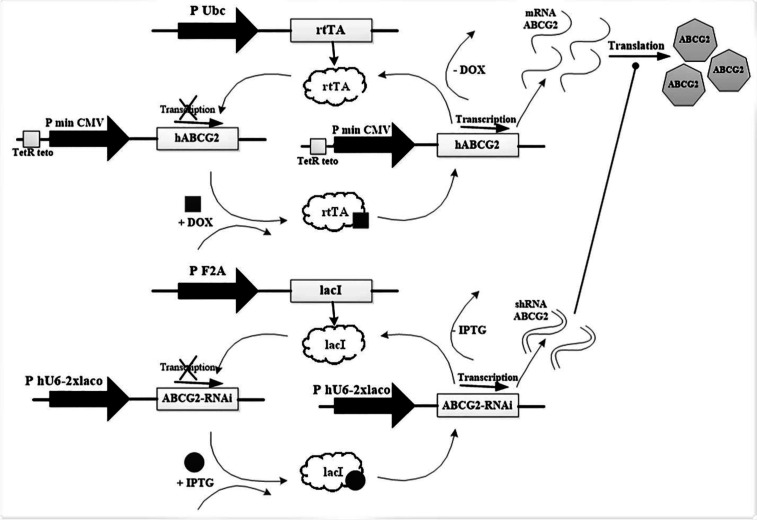
Human ABCG2 and BCRP-shRNA gene maps

In this study, the gene of interest and the corresponding shRNA was cloned into the lentiviral vectors and then packaged into viral particles. In the construct, according to Figure 5, the human BCRP gene was under the control of the promoter Tet (tetracycline-controlled) and the antibiotic selection marker Puromycin was used. Furthermore, BCRP-shRNA gene was under the control of the promoter *β*-galactosidase (controlled by *β*-galactosidase) and the antibiotic selection marker Blasticidine was used.

*Transfection of MCF-7 cancer cells with the lentiviral vectors containing BCRP gene and determination the effective concentration of Puromycin*

MCF-7 cancer cells are purchased from ATCC cell bank and seeded at a concentration of 1 × 10^5^ in a 6 well plate in RPMI1640 medium containing 10% FBS, penicillin (100 IU milliliter-1), and streptomycin (100 milligram milliliter-1). When the cell confluency reaches 70%, transfection is done. 

MCF-7 cells transfected with lentiviral vectors containing BCRP gene were planted at 50 × 10^3^ in a 24-well plate. Then, 7 different concentrations of puromycin antibiotics (0.1, 0.5, 1, 2, 5 and 10 micromolar) were prepared 24, 48, and 72 h after incubation. The cells were inspected each day with an optical microscope and finally, the results of cell toxicity were analyzed. The concentration of 1 micromolar of puromycin antibiotic was selected as the minimum concentration resulting in cell death after 72 h. This minimum concentration is used as a selection factor for MCF-7 cells, that have been transfected with the BCRP gene construct. Typically, when a cell does not receive the BCRP gene construct, it is sensitive to puromycin and will be eliminated. 

*Transfection of specific shRNA and determination the effective concentration of Blasticidine*

MCF-7 cells were plated at 50 × 10^3^ in a 24-well plate and transfected with the lentiviral vector, containing specific shRNA against BCRP. Then, the transfected cells were treated with 7 different concentrations of blasticidine antibiotics (0, 25, 50, 75, 100 and 150 microgrammicrogram) for 24, 48, and 72 h. The cells were inspected each day with an optical microscope and the results were analyzed to define cell toxicity. In this research, the concentration of 75 microgram of blasticidine was selected as the minimum concentration resulting in cell death after 72 h.

This minimum concentration is used as a selection factor for MCF-7 cells, that have been transfected with the shRNA of BCRP gene construct. Typically, when a cell does not receive the shRNA of BCRP gene construct, it is sensitive to blasticidine and will be eliminated.

To study the gene expression at the RNA level, we designed special primers using Gene Runner for BCRP gene and a reference gene (*β*-Actin) according to Table 1. To check the specificity of the primers, we BLASTED the designed primers.

*RNA extraction and real-time RT-PCR*

Total RNA was extracted using RNeasy Mini Kit according to the manufacturer’s instruction (Qiagen) and treated with DNaseI (Fermentas, Lithuania). One microgram of total RNA, MMLV reverse transcriptase (Fermentas, Lithuania) and a random hexamer primer were mixed for cDNA synthesis. Real-time PCR was performed with SYBR green master mix (Takara, Japan) in a Thermal Cycler Rotor-Gene 6000 (Corbett, Australia), according to the manufacturer’s protocol (Takara, Japan). The Expressions were normalized to *β*-Actin level. All experiments were performed in triplicate and all results were analyzed by the ΔΔCt method. 

Data from the cell populations with different treatments were analyzed using multivariate analysis of variance (MANOVA) with Tukey post hoc or student’s *t*-test and a value of *p* < 0.05 was considered statistically significant in the experiment.

*Flow cytometry*

Flow cytometry was used to quantify ABC-transporter in the presence and absence of mitoxantrone. Samples were seeded at a density of 5 × 10^5^ cells/well in 6-well plates. After trypsin incubation, the resuspended cells were divided equally in medium containing BCRP fluorescent substrate, mitoxantrone (3 micromolar) alone or in combination with 200 micromolar novobiocin (the specific BCRP-pump inhibitor). Following a 37 °C incubation for 30 min, the cells were harvested, washed with ice-cold PBS. Next the cell suspensions were divided into two equal fractions, each of which was kept on ice in the dark for analysis of kinetic flow cytometry measurements. The rest of the cells were incubated in a complete medium supplemented or not with the inhibitor, at 37 °C for 1 h. After the treatments, the cells were washed twice with ice cold PBS, and kept in the dark for FACS analyses for efflux kinetic experiments. The samples were analyzed on a Partec^TM^ cytometer equipped with a standard argon laser for 488-nanometer excitation and with 530/30 nm bandpass (FL1) (27). All experiments were performed at least in three independent times. Inhibitory of compounds were calculated from the shift of MFI caused by the tested compound in MCF-7 cells related to the shift of MFI caused by the BCRP pump inhibitor(novobiocin), according to the Equation 1 (28). 

∆EFFLUX=MFITHREATwith novobicin-MFITHREATwithout novobicinMFICRTLwith novobicin-MFICRTLwithout inhibitor

The value of ΔEfflux shows the activity level of the BCRP pump. If its value is greater than one, it indicates an increase in pump activity and more drug withdrawal from the cell. If its value is less than one, it means that the activity of the pump has been repressed and less drug is removed by the pump from the cell.

## Results

*Inducing and silencing of BCRP expression*

**Figure 6:**
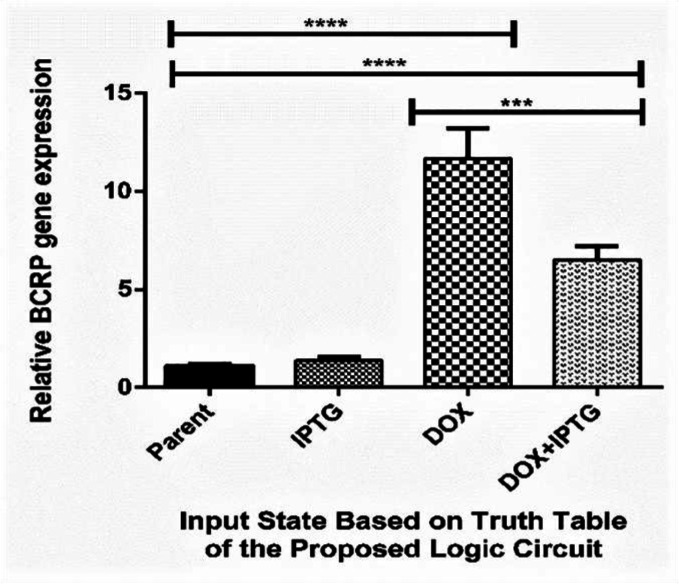
**Relative expression of BCRP in various states in MOI = 5**

The effect of the inducers (doxycycline and IPTG (Isopropyl *β*-*D*-1-thiogalactopyranoside)) on the BCRP gene expression (mRNA) and silencing were investigated by real-time PCR. SB abbreviation has been used for sodium butyrate salt and DOX for doxycycline antibiotics. The results of the Real-Time PCR for MCF-7 cells co-transfected with BCRP and shRNA in MOI = 5 are presented in Figure 6. The columns will show the rate of changes relative to the MCF-7 parent cell and the asterisks will show statistical significance. When doxycycline (2 µicro gram/milliliter) and sodium butyrate (1 millimolar) are present in the medium, expression of the BCRP gene significantly increased.

In the presence of doxycycline, sodium butyrate, and IPTG, the expression of the shRNA was induced and significantly decreased the expression of the gene, Table 2.

According to the Figure 6, the increased expression of the BCRP gene in the transfected cells compared to the parent cells is considered to be HIGH logic (‘1’), while reduction of BCRP gene expression in the presence of IPTG is considered as LOW logic (‘0’). 

*Effect of mitoxantrone and novobiocin treatment on BCRP efflux pump in MCF-7 Cells*

Flow cytometry was used to determine the effect of induction and silencing of the BCRP expression on the efflux of the pump in MCF-7. The pump substrate, which has fluorescent properties, can remain in the cell in the presence of an inhibitor, therefore, more light will come from the cell into the detector. If there is no inhibitor, the substrate is driven by the pump out of the cell and decrease the fluorescent emission from the cell.

**Figure 7:**
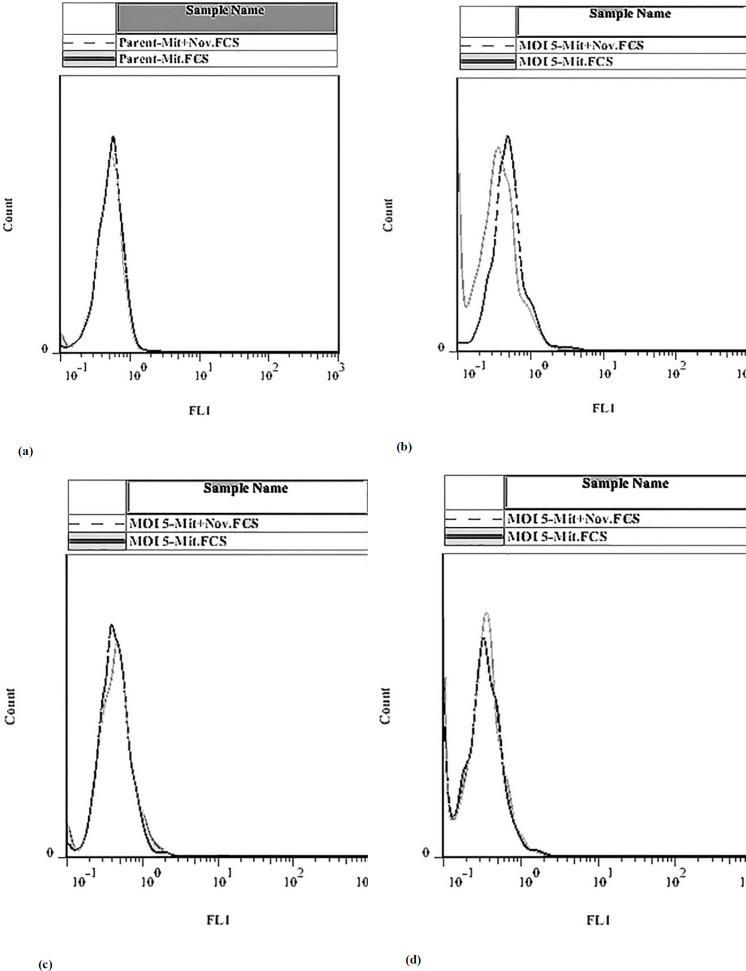
Effect of doxycycline and IPTG on the accumulation of mitoxantrone in MCF-7 cells. After a pretreatment of MCF-7 cells with doxycycline and IPTG, cells were incubated with 3 micomolar of mitoxantrone in the absence or presence of novobiocin. (a) MCF-7 Parent, (b) In the presence of doxycycline and absence of IPTG, (c) In the presence of doxycycline and IPTG, (d) In the presence of IPTG and absence of doxycycline. Concerning the part a, part (b) shows the right shift in the fluorescence peak in doxycycline-treated cells. Furthermore, parts (c and d) show the decrease in the level of BCRP pump in IPTG-treated samples

Our results showed that in the presence of the inhibitor in the Dox-induced BCRP gene expression, the pump was suppressed and the drug remained in the cells. However, when the BCRP gene expression was silenced by the shRNA, there was no significant change in the treated and untreated transfected cells, Figure 7.

**Figure 8:**
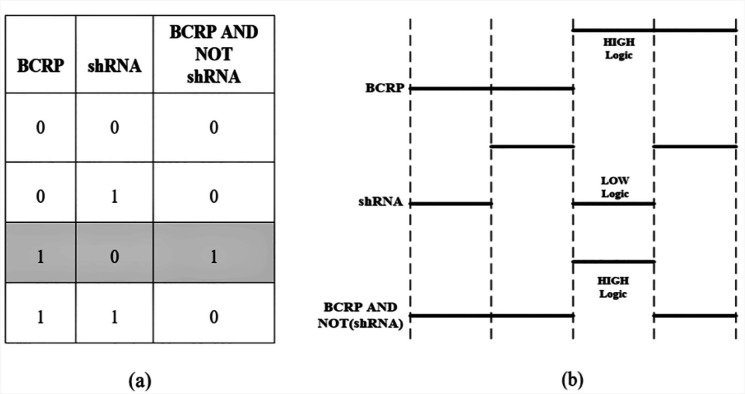
(a) True table of proposed biological circuit based on the data analysis (b) Schematic output of the proposed biological circuit based on the analysis

As a result, the truth table based on the data analysis in the presence of sodium butyrate is in accordance with the functional behavior of the proposed biological circuit which is shown in Figure 8. 

## Discussion

In the present study, we explored the possibility of synthetic biology based on the genetic logic circuit *A* and (not *B*) using the inducible expression of the both BCRP drug resistance pump and its specific shRNA in MCF-7 cancer cells using the third generation of lentiviral vectors. Our predicted output of the proposed circuit confirmed by the results of the Real-Time PCR and flow cytometry at the RNA and protein (functional) levels. At the RNA level, the expression of BCRP gene induced in the presence of doxycycline and silenced in the presence of IPTG due to the expression of the shRNA resulted in the degradation of the mRNA of the BCRP gene. Furthermore, at the protein (functional) level, induction the expression of the BCRP pump resulted in driving out of the substrate (mitoxantrone) from inside the cells leading to the decrease of the fluorescent emission from the transfected cells. On the other hand, induction of the expression of the shRNA abolished the functional state of the pump leading to the accumulation of the substrate (mitoxantrone) inside the cells.

Frezza *et al.* designed a complete set of modular DNA-based Boolean logic gates (AND, OR, and AND-NOT) and demonstrated the Boolean XOR function using suggested biological gates. The function of proposed molecular logic gates confirmed using an input reporter that releases an output fluorescent signals in response to a specific single-stranded DNA inputs (29).

Rinaudo *et al.* use RNA interference in human kidney cells to compose a molecular computing unit that carries out general Boolean logic. The encoding rules, combined with a specific arrangement of the siRNA in a synthetic gene network, which allows calculation of any Boolean expression in standard forms of Boolean logic using siRNAs, directly with up to five logic variables (30).

Culler *et al.* illustrate a class of engineered RNA control device that detects signaling through the nuclear factor signaling pathways in human cells and combines these pathways to produce new behaviors to noninvasive sensing and reprogrammed cellular destinies (31).

Pasotti *et al.* proposed two modular biological systems that could mimic multiplexing and demultiplexing logic functions using logic gates (AND, OR, and NOT) with protein/autoinducer or protein/DNA interactions and interconnecting them to create the final circuits. They showed that part of the implemented logic functions has been tested in different experiments (32).

Xie *et al*. proposed the classifier circuit (as a Boolean logic circuit) output of which acts as a marker for the detection of tumor cells (HELA from non-HELA types). As a scalable transcriptional/post transcriptional synthetic circuit, the selective classifier circuit takes in expression levels of a customizable set of endogenous microRNAs and triggers a cellular reaction only if the expression levels match a predetermined profile of interest (10). 

Auslander *et al*. designed a set of synthetic transcription-translation control devices. They show the digital functions of AND, NOT, and NAND gates, in a mammalian cell. With these three gates, they were able to perform calculations by half-subtraction and a half-adder for cells (33).

**Table 1:** Primer Sequences and amplicon size for Real-Time RT-PCR

**Amplicon Size (bp)**	**Sequences**	**Genes**
150	Forward, 5′-TCATGAAGTGTGACGTGGACATC-3′ Reverse, 5′-CAGGAGGAGCAATGATCTTGATCT-3′	*β*-Actin
150	Forward, 5'- GTTTCAGCCGTGGAAC-3' Reverse, 5'-CTGCCTTTGGCTTCAAT-3'	BCRP

**Table 2:** **Reduction in gene expression after shRNA induction**

***P(H)***	***R***	**Sample2**	**Sample1**
0.000 DOWN	0.98	Dox+SB	IPTG
0.000 DOWN	0.7	DoX+SB	Dox+SB+IPTG

## Conclusion

In conclusion, we successfully designed and implemented the genetic logic circuit *A *and (not *B*) using the inducible expression of the both BCRP drug resistance pump and its specific shRNA in MCF-7 cancer cells using the third generation of lentiviral vectors. The output of the proposed circuit was confirmed at both RNA and protein (functional) levels.

At the RNA level, the effect of the inducers on the BCRP gene expression and silencing were investigated by real-time PCR. Furthermore, at the protein level, the induction of the expression of the BCRP pump resulted in driving out of the substrate from inside the cells leading to the decrease of the fluorescent emission from the transfected cells.
